# Lipidomic Assessment of Plasma and Placenta of Women with Early-Onset Preeclampsia

**DOI:** 10.1371/journal.pone.0110747

**Published:** 2014-10-17

**Authors:** Henri Augusto Korkes, Nelson Sass, Antonio F. Moron, Niels Olsen S. Câmara, Tatiana Bonetti, Ana Sofia Cerdeira, Ismael Dale Cotrim Guerreiro Da Silva, Leandro De Oliveira

**Affiliations:** 1 Department of Obstetrics – Federal University of Sao Paulo, Sao Paulo, Sao Paulo, Brazil; 2 Laboratory of Clinical and Experimental Investigation – School Maternity Vila Nova Cachoeirinha, Sao Paulo, Sao Paulo, Brazil; 3 Department of Immunology – University of Sao Paulo, Sao Paulo, Sao Paulo, Brazil; 4 Department of Gynecology - Federal University of Sao Paulo, Sao Paulo, Sao Paulo, Brazil; 5 Department of Medicine, Beth Israel Deaconess Medical Center, Harvard Medical School, Boston, Massachusetts, United States of America; INRA, France

## Abstract

**Introduction:**

Adipose tissue is responsible for triggering chronic systemic inflammatory response and these changes may be involved in the pathophysiology of preeclampsia.

**Objective:**

To characterize the lipid profile in the placenta and plasma of patients with preeclampsia.

**Methodology:**

Samples were collected from placenta and plasma of 10 pregnant women with preeclampsia and 10 controls. Lipids were extracted using the Bligh–Dyer protocol and were analysed by MALDI TOF-TOF mass spectrometry.

**Results:**

Approximately 200 lipid signals were quantified. The most prevalent lipid present in plasma of patients with preeclampsia was the main class Glycerophosphoserines-GP03 (PS) representing 52.30% of the total lipid composition, followed by the main classes Glycerophosphoethanolamines-GP02 (PEt), Glycerophosphocholines-GP01 (PC) and Flavanoids-PK12 (FLV), with 24.03%, 9.47% and 8.39% respectively. When compared to the control group, plasma samples of patients with preeclampsia showed an increase of PS (p<0.0001), PC (p<0.0001) and FLV (p<0.0001). Placental analysis of patients with preeclampsia, revealed the PS as the most prevalent lipid representing 56.28%, followed by the main class Macrolides/polyketides-PK04 with 32.77%, both with increased levels when compared with patients control group, PS (p<0.0001) and PK04 (p<0.0001).

**Conclusion:**

Lipids found in placenta and plasma from patients with preeclampsia differ from those of pregnant women in the control group. Further studies are needed to clarify if these changes are specific and a cause or consequence of preeclampsia.

## Introduction

Preeclampsia is a systemic disease characterized by intense inflammatory response, endothelial injury, platelet aggregation, coagulation system activation and increase vascular resistance. It affects about 5–8% of all pregnant women [1]–[3]. The diagnosis of preeclampsia is based on the development of hypertension (≥140/90 mmHg) and significant proteinuria (≥300 mg/24 hours) after 20 weeks of gestation [4].

The systemic complications of preeclampsia are not limited to the gestational period and recent studies have shown long-term adverse outcomes, such as increased risk for developing chronic hypertension, ischemic heart disease, acute myocardial infarction and venous thromboembolism, requiring longer follow-up and surveillance of these patients throughout their lives [4], [5]. Despite its relevance, preeclampsia pathogenesis is not completely understood. It has been established that the trophoblast has a key role in this process and many other conditions related to chronic inflammation can be relevant in different stages of the disease [3].

### Obesity and Preeclampsia

Obesity, defined by the World Health Organization (WHO) through the body mass index above 30 kg/m^2^, is a growing epidemic problem and it affects 500 million adults across the world [6], [7]. It represents an important health problem and it has an enormous impact on modern obstetrics.

Adipose tissue is responsible for triggering chronic systemic inflammatory response, with increased levels of inflammatory cytokines such as TNF-α, IL-6 and MCP-1. The inflammatory response related to obesity has been considered as the link between this condition and preeclampsia [6], [8]–[13].

Although the link between obesity and inflammatory response is well recognized, the roles of lipids in the cell function are even more extended. These molecules are responsible for the control of important cellular processes, including proliferation, apoptosis, metabolism and migration. They also assist in the transmission of biological information across cell membranes, directly contributing to proper cell functioning [14]–[16]. An impairment in lipid signaling pathways may contribute to the progression of chronic inflammatory diseases, such as autoimmune, allergic, neoplastic, atherosclerosis, hypertension, myocardial hypertrophy and metabolic degenerative diseases [17], [18] and may be also related to preeclampsia pathophysiology.

Lipid molecules are defined by the International Committee for the Classification and Nomenclature of Lipids (ICCNL) in eight categories, based on their chemical functions: Fatty Acyls (FA), Glycerolipids (GL), Glycerophospholipids (GP), Sphingolipids (SP), Sterol Lipids (ST), Prenol Lipids (PR), Saccharolipids (SL) and Polyketides (PK). Each category is further subdivided into lipid main classes and subclasses [19], [20].

Historically, the study of the function and properties of lipids was always extremely complicated due to their structural diversity and large number of isomorphic species. Technically, the distinction between pathogenic and nonpathogenic lipid molecules represents a challenge that has become possible through lipidomics [18], [21], [22].

### Lipidomic analysis

Lipidomic analysis is a global characterization of all kinds of lipid molecules in biological system. The methodology used is mass spectrometry (MS) [22]–[24]. A technique known as Matrix-Assisted Laser Desorption/Ionization - Mass Spectrometry (MALDI-MS), has been the preferred method to evaluate lipidomics because it is relatively easy to handle [25], [26]. MALDI is an ionization technique enabled by a laser beam (light amplification by stimulated emission of radiation) that acts upon a sample mixed with a matrix. This process generates ionized molecules. For complete separation, the most widely used technology is the time of flight (TOF), which consists of a long pipe (tube flight) capable of separating the ionized molecules according to a ratio of mass to charge (m/z) [25].

Lipidomic analysis in preeclampsia is a new research line. Recently, we demonstrated that women with early-onset preeclampsia have particular lipids in their plasma when compared to those with healthy pregnancy [26]. Additionally, Baig et al. published their findings evaluating samples of syncytiotrophoblast microvesicles from human placenta [27]. These authors also demonstrated that there was a significant increase of some classes of lipids as well as a reduction of others in samples from preeclamptic women.

Given the strong association between obesity and early dyslipidemia with preeclampsia and the first reports associating distinct lipid species with the disease, this study aimed to find a specific lipid profile that may be characteristic for these patients. Here we evaluated plasma samples and placental tissues of women with early-onset preeclampsia and established an interesting panel of lipids in these different settings.

## Material and Methods

### Ethics Statement

All participants in this study have provided their written informed consent. This study was approved by the Research Ethics Committee of the Federal University of São Paulo with the number 297/027 and by the Research Ethics Committee of the School Maternity Vila Nova Cachoeirinha with the number 34/2011, linked with the National database (CEP/CONEP) under the protocol number CAAE - 18100813.1.0000.5505.

### Study Population

This is a case-control study involving 20 pregnant women (10 women with early-onset preeclampsia and 10 women with healthy pregnancy). All samples were collected at the School Maternity Vila Nova Cachoeirinha from October 2011 to April 2013. Early-onset preeclampsia was defined as blood pressure ≥140/90 mmHg and significant proteinuria (≥300 mg/24 hours) after 20 weeks of gestation and before 34 weeks.

### Sample collection

#### Blood

Five milliliters of peripheral blood were drawn in EDTA-tube at the time of delivery. Immediately after collection, blood samples were centrifuged at 2000 rpm for 5 minutes and supernatants aliquoted and stored at −80°C for subsequent lipid extraction.

#### Placenta

Immediately after cesarean delivery, one fragment was removed from the central region of the basal plate of the placenta, with a wedge shape of approximately 3.0 cm in diameter at its greatest diameter, obtained with a sterile scalpel blade N°15. This fragment was divided in 3 smaller pieces of 1×1 cm, washed in saline solution and immediately frozen and stored at −80°C for further processing and lipid extraction. For lipid extraction the frozen placental sample of 1×1 cm was plunged into liquid nitrogen for 1 minute and crushed using marble stone until obtaining small fragments (powder). Powder was then placed in a dry tube containing 300 µl of Milli-Q water. This material was subjected to further mixing and homogenization in a mechanical processor for five minutes and the resulting material subjected to lipid extraction.

### Lipid extraction

Lipids were extracted from each sample using the Bligh–Dyer protocol [28]. Immediately after thawing, each 50 µL of plasma and placental homogenated (described above) were dissolved in a mixture of chloroform–methanol (125∶250 µL) and vortexed well. After vortexing, 125 µL of chloroform and 100 µL of deionized water were added to supernatant and centrifuged at 1000 rpm in a table-top centrifuge for 5 min at room temperature. Following this protocol a two-phase system (aqueous top, organic bottom) was achieved. The bottom phase containing lipids was gently recovered using a Pasteur pipette; they were dried and sealed to be stored at −80°C.

### Reagents

All chemicals were of analytical reagent grade and they were used as received. Chloroform (CHCl3) and methanol (MeOH) were purchased from Burdick & Jackson (Muskegon, MI, USA). 2,5-Dihydroxybenzoic acid (DHB) was purchased from ICN Biomedicals (Aurora, OH, USA). Distilled water was deionized on a Millipore Milli-Q water reagent system (Millipore, Bedford, MA, USA). EDTA-tubes were purchased from Sigma-Aldrich (St. Louis, MO).

### Mass spectrometry analysis

MALDI-MS spectra were acquired in the positive ion and reflector modes using the equipment MALDI TOF-TOF - Ultraflex model - Bruker and matrix 2,5 - DHB - White (2,5-Dihydroxy benzoic acid - 40 mg/ml in acetonitrile). The main operating condition used was 10 V (sample plate). The laser irradiation consisted of diverse shots during 60–90 seconds in the region where the sample had been placed.

### Data processing

Raw data were analyzed by MarkerLynx (Waters, UK) for peak detection and alignment. The parameters were set as follows: mass tolerance was set at 100 ppm (suggested more than twice the instrument mass accuracy considering extreme value in signal saturation condition); peak width at 5% height and peak-to-peak baseline noise were calculated automatically by the software; mass window was set at 0.1 amu (atomic mass units); retention time window was set at 1 min (considering the maximum variation obtainable by a CapLC system); noise elimination level was 5; minimum intensity was set at 5%; peak intensity and retention time were normalized with the signal of the internal standard. This procedure allowed deconvolution alignment, and data reduction to give a table of mass and relative retention time pairs with associated relative intensities for all the detected peaks. Then data matrix was exported for partial least squares discriminant analysis (PLS-DA). In order to find differential circulating lipids, a VIP parameter (Variable Importance in the Projection) was employed to reflect the variable importance in the discriminant analysis. The major discriminant variables returned by the PLS-DA model were selected and underwent the Mann–Whitney U test to confirm the differential expression between groups. Those peaks showing p<0.05 were considered as having statistically significant differences.

### Statistical Analysis

The lipid composition of the samples was established by the area of the peaks obtained for the main lipids identified. The data were normally distributed and statistical analysis was performed using the Student's t-test, using the GraphPad Prism version 6. Differences between the groups were considered statistically significant when p<0.05.

## Results

**Table 1** shows the demographic and obstetric characteristics of study participants.

**Table 1:** Demographic and obstetric characteristics of study subjects.

	Control	Preeclampsia	P
	(n = 10)	(n = 10)	
Maternal age (years)	25.3±6.237	22.4±7.152	0.372
Gestational age (weeks)	38.6±1.174	35.5±3.504	0.026
Weight gain (Kg)	10.77±5.094	20.22±5.037	0.001
BMI (Kg/m2)	22.59±6.912	26.73±5.379	0.154
Blood Pressure			
Systolic (mmHg)	116±10.666	140.4±4.971	<0.0001
Diastolic (mmHg)	70.6±7.306	91±5.436	<0.0001
Birth Weights (g)	3190.6±581.115	2607±881.328	0.0975
Proteinuria (mg)	NE	1624.4±1072.915	NE

Results are expressed as mean ± standard deviation. Significant at P<0.05.

BMI – Body Mass Index.

NE - not evaluated.

### Mass Spectrometry

**Figure 1:**
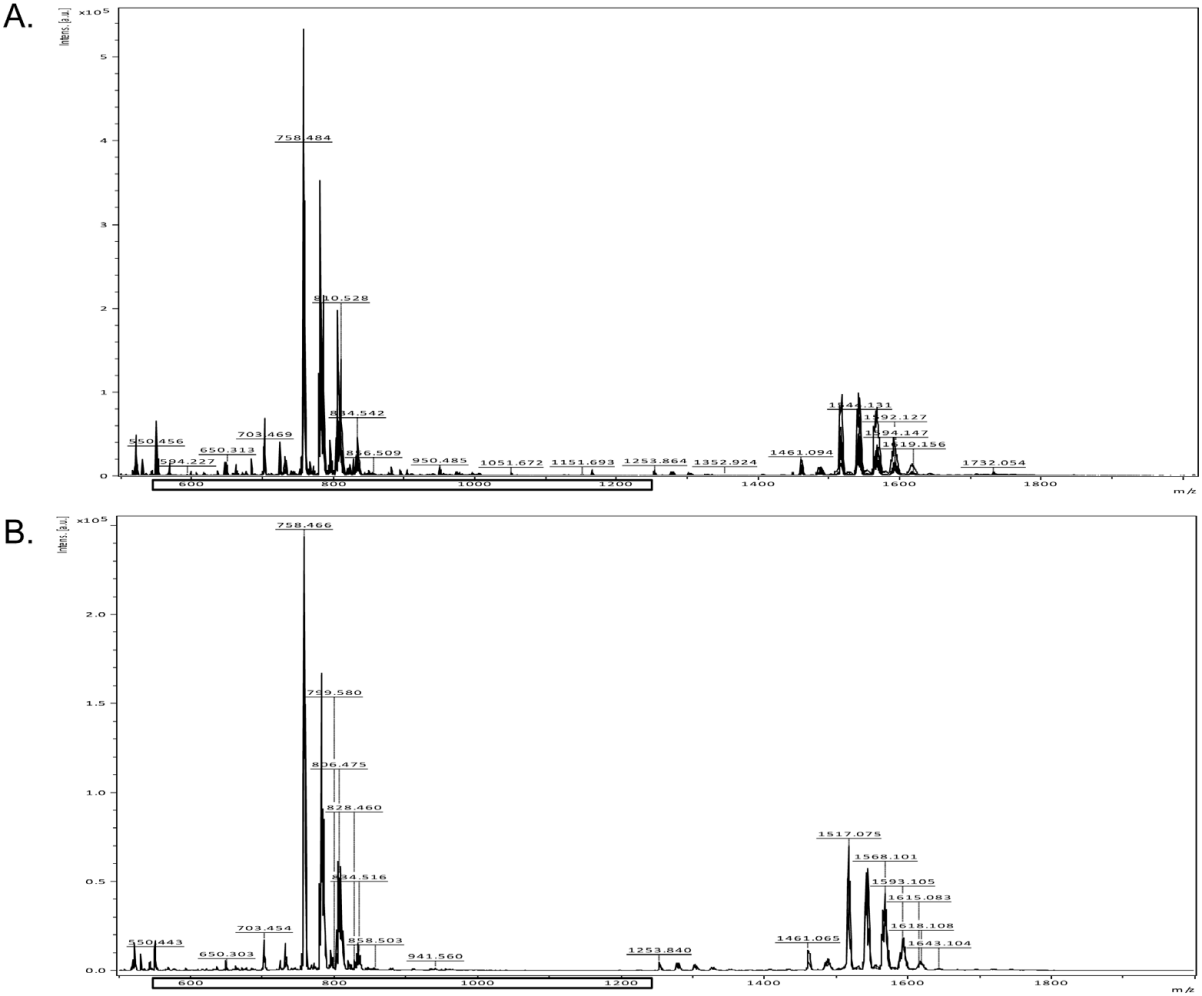
Representative lipid spectrum showing signals in plasma samples of control and preeclamptic patients. The signals were identified between the lipid tracks acquisition from 600 to 1200 m/z.

Approximately 200 signals were identified between the lipid tracks acquisition from 600 to 1200 m/z (**Figure 1**). The identification of the different lipids found was carried out through the Lipid Database Search (http://www.lipidmaps.org), using the results of m/z analyzes.

### Plasma samples

**Figure 2:**
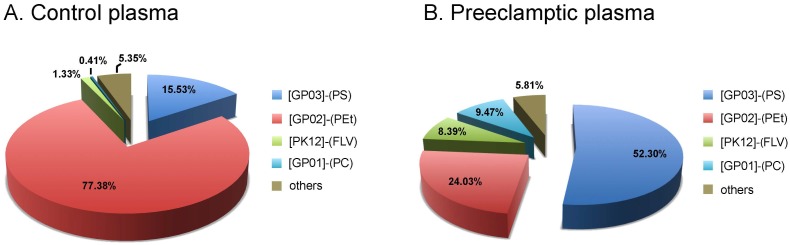
Lipid composition detected in plasma from control and preeclamptic patients. Comparison of relative distribution of main class of lipids in plasma of normal (A) and preeclamptic (B) patients, established by the area of the peaks obtained for the main lipids identified. N = 10 in each group. [GP03]-(PS): Glycerophosphoserines or Phosphatidylserines; [GP02]-(PEt): Glycerophosphoethanolamines or Phosphatidylethonolamines; [PK12]-(FLV): Flavonoids; [GP01]-(PC): Glycerophosphocholines or Phosphatidylcholines.

**Table 2** and **Figure 2** show the lipid composition found in plasma samples from both groups. Plasma analysis of patients with preeclampsia revealed the main class of Glycerophosphoserines-GP03 (PS) as the most prevalent lipid, representing 52.30% of the total lipid composition, followed by Glycerophosphoethanolamines-GP02 (PEt), Glycerophosphocholines-GP01 (PC) and Flavanoids-PK12 (FLV). When compared to the control group, plasma samples of patients with preeclampsia showed an increased proportion of PS (p<0.0001), PC (p<0.0001) and FLV (p<0.0001).

**Table 2:** Main classes of lipids in plasma samples.

Plasma	Group		P
[Main class]-(Common name)	Control (n = 10)	Preeclampsia (n = 10)	
Glycerophosphocholines [GP01]-(PC)	0.41%	9.47%	<0.0001
Glycerophosphoethanolamines [GP02]-(PEt)	77.38%	24.03%	<0.0001
Glycerophosphoserines [GP03]-(PS)	15.53%	52.30%	<0.0001
Glycerophosphoglycerols [GP04]-(PG)	3.14%	0.90%	0.003
Glycerophosphoinositols [GP06]-(PI)	0.62%	0.13%	NS
Glycerophosphates [GP10]-(PAc)	0.09%	1.40%	<0.0001
CDP-Glycerols [GP13]	0.02%	0.00%	NE
Triradylglycerols [GL03]-(TG)	0.22%	0.16%	NS
Phosphosphingolipids [SP03]-(SM)	0.00%	0.37%	<0.0001
Neutral glycosphingolipids [SP05]	0.00%	1.72%	<0.0001
Sterols [ST01]	0.31%	0.03%	<0.0001
Steroid conjugates [ST05]	0.00%	0.65%	0.0002
Polyprenols [PR03]	0.02%	0.00%	NE
Acylaminosugars [SL01]	0.00%	0.21%	NS
Other acyl sugars [SL05]	0.38%	0.00%	<0.0001
Flavonoids [PK12]	1.33%	8.39%	<0.0001
Fatty esters [FA07]	0.55%	0.24%	NS

Results are expressed as percentage. Significant at P<0.05.

NE - not evaluated.

NS – not significant.

Common names used: (PC) Phosphatidylcholines, (PEt) Phosphatidylethonolamines, (PS) Phosphatidylserines, (PG) Phosphatidylglycerols, (PI) Phosphatidylenositos, (PAc) Phosphatidic acid, (TG) Triradylglycerols, (SM) Sphingomyelins.

Although in smaller proportion, other increased lipids in patients with preeclampsia were Glycerophosphates-GP10 (PAc) (p<0.0001), Phosphosphingolipids-SP03 (SM) (p<0.0001), Neutral glycosphingolipids-SP05 (p<0.0001) and Steroid conjugates-ST05 (p<0.0002). The main class PEt, was reduced in patients with preeclampsia when compared to the control group (p<0.0001). Other main lipid classes reduced in preeclampsia group were: Glycerophosphoglycerols-GP04 (PG) (p<0.003), Sterols-ST01 (p<0.0001) and Other acyl sugars-SL05 (p<0.0001).

### Placental samples

**Figure 3:**
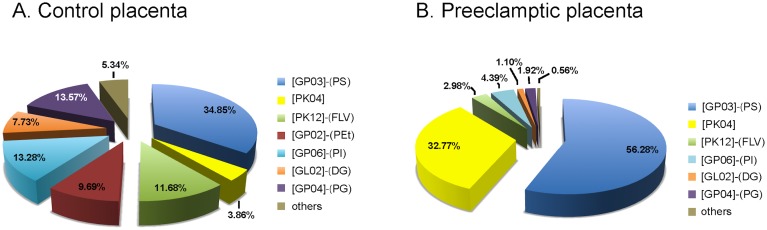
Lipid composition detected in placenta from control and preeclamptic women. Comparison of relative distribution of main class of lipids in placenta of normal (A) and preeclamptic (B) patients, established by the area of the peaks obtained for the main lipids identified. N = 10 in each group [GP03]-(PS): Glycerophosphoserines or Phosphatidylserines; [PK04] =  Macrolide and lactone polyketides; [PK12]-(FLV): Flavonoids; [GP02]-(PEt): Glycerophosphoethanolamines or Phosphatidylethonolamines; [GP06]-(PI): Glycerophosphoinositols or Phosphatidylinositols; [GL02]-(DG): Diradylglycerols; [GP04]-(PG): Glycerophosphoglycerols or Phosphatidylglycerols.

**Table 3** and **Figure 3** show the lipid composition found in placental samples from both groups. The placental analysis of patients with preeclampsia revealed the main class PS as the most prevalent lipid, representing 56.28% of the total composition. Other main classes found were: Macrolides and lactone polyketides-PK04 with 32.77%, both were increased in preeclamptic placentas when compared to the control group, PS (p<0.0001) and PK04 (p<0.0001). Some lipids found in placentas from patients with preeclampsia were reduced when compared to control group; PEt (p<0.0001), PG (p<0.0001), Glycerophosphoinositols-GP06 (PI) (p<0.0001), Glycerophosphoinositol monophosphates-GP07 (p<0.0001), Diradylglycerols-GL02 (p<0.0001), Triradylglycerols-GL03 (p<0.0001), Acidic glycosphingolipids-SP06 (GM3) (p<0.0001), Steroid conjugates-ST05 (p<0.0001), Other acyl sugars-SL05 (p<0.0001) and Flavonoids-PK12 (p<0.0001).

**Table 3:** Main classes of lipids in placental samples.

Placenta	Group		P
[Main class]-(Common name)	Control (n = 10)	Preeclampsia (n = 10)	
Glycerophosphocholines [GP01]-(PC)	0.00%	0.02%	NE
Glycerophosphoethanolamines [GP02]-(PEt)	9.69%	0.00%	<0.0001
Glycerophosphoserines [GP03]-(PS)	34.85%	56.28%	<0.0001
Glycerophosphoglycerols [GP04]-(PG)	13.57%	1.92%	<0.0001
Glycerophosphoinositols [GP06]-(PI)	13.28%	4.39%	<0.0001
Glycerophosphoinositol monophosphates [GP07]	0.57%	0.00%	<0.0001
Glycerophosphates [GP10]-(PAc)	1.30%	0.24%	NS
Diradylglycerols [GL02]-(DG)	7.73%	1.10%	<0.0001
Triradylglycerols [GL03]	1.04%	0.08%	<0.0001
Neutral glycosphingolipids [SP05]	0.07%	0.00%	NE
Acidic glycosphingolipids [SP06]- (GM3)	0.26%	0.06%	<0.0001
Sterols [ST01]	0.00%	0.12%	NS
Steroid conjugates [ST05]	1.68%	0.00%	<0.0001
Other acyl sugars [SL05]	0.42%	0.04%	<0.0001
Flavonoids [PK12]	11.68%	2.98%	<0.0001
Macrolide lactone polyketide [PK04]	3.86%	32.77%	<0.0001

Results are expressed as percentage. Significant at P<0.05.

NE - not evaluated; NS – not significant.

Common names used: (PC) Phosphatidylcholines, (PEt) Phosphatidylethonolamines, (PS) Phosphatidylserines, (PG) Phosphatidylglycerols, (DG) Diradylglycerols; (PI) Phosphatidylenositos, (PAc) Phosphatidic acid, (TG) Triradylglycerols, (SM) Sphingomyelins, (GM3) Gangliosides.

## Discussion

The pathogenesis of preeclampsia has its roots on deficient trophoblastic invasion and failure in spiral artery remodeling. This incomplete transformation of the spiral arteries leads to inadequate placental perfusion and consequently to placental oxidative stress [29]. The altered placenta then releases great amount of microparticles, debris and antiangiogenic factors into the maternal circulation [30]–[32]. All these factors are supposed to act in synergy to initiate and to maintain an intense inflammatory response and an antiangiogenic state.

Maternal obesity has been considered to have important impact on the genesis of preeclampsia as obese women have higher risk for developing the disease. In addition, women with body-mass-index lower than 20 have lower risk to develop preeclampsia [33]. The complete link between preeclampsia and obesity has not been defined. However, it is known that the inflammatory aspect that characterize the lipotoxicity of adipose tissues leads to maternal endothelial dysfunction, decreases trophoblastic invasion and influences placental metabolism and function. In addition, the chronic inflammatory response of the “metabolic syndrome” can contribute to the systemic inflammation seen in preeclampsia [13].

Actually, lipids play important roles in cellular function as they are the main components of biological membranes [14]. Therefore, lipids can participate in the constitution of membrane receptors, ion channels and in cell signaling mechanisms. In addition, many lipids can act as endogenous ligands, binding to specific receptors and then initiating several immunological responses [34].

In this study we investigated and established the main composition of the lipid profile identified in plasma and placental tissue of normal pregnant and preeclamptic women. Either placental tissues or plasma from patients with preeclampsia expressed different lipid profile when compared to normal pregnant women. These findings suggest that specific lipid species may be more associated with risk of developing preeclampsia than others. Additional functional studies will be necessary to clarify the involvement of these lipids in the pathophysiology of preeclampsia.

*PS* were the most prevalent species of lipids in preeclamptic women group. These lipids belong to Glicophospholipids category, representing the major lipid constituent of cell membranes and lipoproteins. They play different biological roles, acting as signaling molecules involved in the processes of oxidative stress, apoptosis and coagulation, all exacerbated in preeclampsia [35]–[39].

*PC species* also belong to Glicophospholipids category and were increased in plasma of patients with preeclampsia. *PC* species are precursors of several molecules that act as lipid second messengers, including phosphatidic acid. Increased levels of *PC* have been associated with increased cell proliferation. These lipids have been recently correlated with different tumor behaviors and cancer progressions. They are probably important for treatment considerations [40]–[42].

Oxidative stress generally affects lipid function due to changes in their native behavior. It can generate numerous different lipids that have diverse biological activities [43], [44]. Glicerophospholipids, when oxidized, induce platelet aggregation, monocyte adhesion to endothelial cells, present in atherosclerotic lesions and they play an important role in signaling inflammatory response [45], [46]. Thus, this increase in PS and PC in preeclampsia suggests a role in the inflammatory and oxidative phenomena observed in these patients.

There was a curious reduction of PEt in plasma samples of the preeclampsia group, compared to the control group. Apparently the reduction of PEt in the endoplasmic reticulum is associated with araquidonic acid release [47], [48]. This is the precursor of prostaglandins, thromboxanes and prostacyclins by the cyclooxygenase pathway and leukotrienes by the lipoxygenase pathway. Prostaglandins cause vasodilation, inhibition of platelet aggregation and pain. Thromboxane A2 promotes vasoconstriction and platelet aggregation. It is possible that these lipids act in opposing mechanisms in different patients.

Although also associated with apoptotic processes, cell proliferation and differentiation, the PI lipid class was found in small amounts in the plasma lipid composition in both groups. The SM lipid species, found only in plasma samples from patients with preeclampsia, act in vascular reactivity and mediate cell growth due to intrinsic properties of these vasoactive species [49]. Experiments on hypertensive mice have showed significant increase in SM [50]. Apparently it is involved in processes of endothelial dysfunction, increased production of angiotensin II, elevated levels of thromboxane A2 and hence hypertensive disorders, which could explain its exclusively occurrence in the population of patients with preeclampsia [50]–[52]. The predominant lipid in preeclampsia placental samples was PS, and this aspect can be correlated with the oxidation process.

The Flavonoids are known for their antioxidant properties [53], [54]. These lipids are not present in mammalian lipid composition, and their presence in our study is probably derived from the diet. These lipids interact in the signaling pathways of apoptotic processes, operating in both promotion and inhibition [55], [56]. Evidences support the Flavonoids as protective factors against cardiac ischemic [57], [58]. In our study, we found a reduction in the amount of Flavonoids in samples of placentas from patients with preeclampsia when compared to controls. This did not happen when we performed the analysis of plasma samples that identified an increase of these lipids in patients with preeclampsia.

The Macrolides polyketides-lactone-PK04 have shown greater incidence in placental samples of preeclampsia group. They are not a natural part of mammalian lipid composition and they can be found in bacteria, fungi and plants. Rapamycin, also called Sirolimus, is an important and known polyketide with many biological and pharmacological activities, including antifungal, immunosuppressive, antitumor, neuroprotective and antiaging activities [59]–[63]. Recently, rapamycin has attracted interest for the clinical treatment of organ transplant rejection and autoimmune diseases [64].

Rapamicyn use has been associated with the development or exacerbation of proteinuria [65]–[67]. The pathogenesis of proteinuria is likely multifactorial and may involve tubular and glomerular contributions. Recent data derived from biopsy sub studies of clinical trials, which compared cyclosporine with rapamycin, demonstrated that rapamycin use is associated with tubular damage and tubular proteinuria [68]. In the glomerular compartment, other ones have demonstrated reduced nephrin expression [69] and reduced VEGF, particularly in patients with significant proteinuria [70], [71].

The immunosuppressive effects of rapamycin result from its ability to inhibit proliferation by interfering with the function of the mammalian target of rapamycin (mTOR) [72]. mTOR is a serine/threonine protein kinase which controls the cellular processes of growth, proliferation, transcription, protein biosynthesis and ribosomal biogenesis [59], [73]. mTOR exists in two distinct protein complexes referred to as mTOR complex 1 and mTOR complex 2. The inactivation of mTOR complex 1 kinase activity by rapamycin results in the inhibition of the activities of ribosomal S6 kinase and the eukaryotic translation initiation factor 4E-binding proteins, which have roles in ribosome biogenesis and protein translation, respectively. In contrast, apoptosis and autophagy are also stimulated by rapamycin [72], [74].

Studies in immortalized cell lines originating from human trophoblast suggest a key role for mTOR in the regulation of trophoblast proliferation and it is suggested that the mTOR pathway is a regulator of invasive trophoblast differentiation [75], [76]. In the mature placenta mTOR is expressed at the mRNA level, however, the cellular localization of mTOR and the functional role of this signaling pathway in the placenta after implantation and early placental development remains unknown [75], [77], [78].

Our study did not include other groups, such as gestational hypertension or intrauterine growth restriction, which would be important to evaluate the specificity of this association. In addition it would be interesting to compare the lipid profile between obese pregnant patients with normal outcomes and patients with preeclampsia with and without obesity to better understand if the lipid changes are a reflection of obesity, increased BMI or true PE.

### Conclusion

We identified a different pattern of lipids and distinct concentrations of some lipid species in plasma and placenta samples of preeclamptic patients. Further studies are needed to clarify if these changes are specific to preeclampsia and whether and how they could be related to its pathogenesis.
